# Estimation of cell-free fetal DNA fraction from maternal plasma based on linkage disequilibrium information

**DOI:** 10.1038/s41525-021-00247-z

**Published:** 2021-10-12

**Authors:** Jia Ju, Jia Li, Siyang Liu, Haiqiang Zhang, Jinjin Xu, Yu Lin, Ya Gao, Yulin Zhou, Xin Jin

**Affiliations:** 1 College of Life Sciences, University of Chinese Academy of Sciences, 100049 Beijing, China; 2 BGI-Shenzhen, 518083 Shenzhen, Guangdong China; 3 BGI-genomics, BGI-Shenzhen, 518083 Shenzhen, Guangdong China; 4 United Diagnostic and Research Center for Clinical Genetics, Women and Children’s Hospital, School of Medicine & School of Public Health, Xiamen University, 361102 Xiamen, Fujian China; 5 School of Medicine, South China University of Technology, 510006 Guangzhou, Guangdong China; 6 Guangdong Provincial Key Laboratory of Human Disease Genomics, Shenzhen Key Laboratory of Genomics, BGI-Shenzhen, 518083 Shenzhen, China

**Keywords:** Aneuploidy, Disease prevention, Genetic testing

## Abstract

Cell-free fetal DNA fraction (FF) in maternal plasma is a key parameter affecting the performance of noninvasive prenatal testing (NIPT). Accurate quantitation of FF plays a pivotal role in these tests. However, there are few methods that could determine FF with high accuracy using shallow‐depth whole‐genome sequencing data. In this study, we hypothesized that the actual FF in maternal plasma should be proportional to the discrepancy rate between the observed genotypes and inferred genotypes based on the linkage disequilibrium rule in certain polymorphism sites. Based on this hypothesis, we developed a method named Linkage Disequilibrium information-based cell-free Fetal DNA Fraction (LDFF) to accurately quantify FF in maternal plasma. This method achieves a high performance and outperforms existing methods in the fetal DNA fraction estimation. As LDFF is a gender-independent method and developed on shallow-depth samples, it can be easily incorporated into routine NIPT test and may enhance the current NIPT performance.

## Introduction

Cell-free fetal DNA (cffDNA) in maternal peripheral blood, discovered by Lo et al.^1^, makes it possible to infer the fetal inheritance in a noninvasive way and led to the development of noninvasive prenatal applications, such as fetal sex determination for sex-linked disorders^2^, detection of fetal chromosomal abnormalities^3,4^, and detection of monogenic diseases^5–8^. Compared with the traditional methods for fetal trisomy screening by the measurement of nuchal translucency and biochemical analytes, noninvasive prenatal testing (NIPT) using cffDNA in maternal blood can be non-invasively performed at early trimester with lower false positive rates^9–11^. In the clinical practice of NIPT, the concentration of cell-free DNA (cfDNA) of fetal origin circulating in maternal plasma, referred to as fetal fraction (FF), is a fundamental parameter in the accurate and robust measurement of fetal trisomy. Currently, the minimum FF required for a reliable NIPT result is ~4% due to the fact that low concentration of fetal cfDNA in maternal plasma may cause a false negative result^12,13^. Thus, accurate determination of FF is critical to NIPT performance.

To date, many approaches have been proposed for the determination of FF in maternal plasma. The methods based on the reads or genetic markers from chromosome Y^14–17^ are accurate and direct, but they are only applicable to pregnancies with male fetuses. Several gender-independent methods rely upon the differential patterns between the maternal and fetal cfDNA, such as the distribution difference of fragment length^18^, DNA methylation difference^19,20^, and the fetal-specific alleles^5,21–24^. However, these methods need additional laboratory test and are not cost-effective for practical use. Recently, three kinds of methods have been developed to estimate the FF independent of fetal gender without additional data. Given the fact that fetal cfDNA nonuniformly distributes across the genome relative to maternal cfDNA, the method SeqFF^25^ uses regional read counts to estimate FF. But this method might not be robust for predicting low FF. Another method calculates the distribution of reads starting around nucleosome positions based on the different DNA digestion between fetal and maternal cfDNA^26^, but its accuracy is relatively poor^27^. The third one makes use of the heterozygosity of single nucleotide polymorphisms (SNPs), however its accuracy might be limited for samples with sequencing depth <0.5x^28^.

The maternal plasma of pregnancies contains fetal-specific haplotypes inherited from the father. We assume that the allele types on one of three haplotypes in certain sites might be rectified by imputation process under the assumption that the analyzed samples are diploid, the probability of which should be correlated with the FF in maternal plasma. We confirmed this hypothesis and trained a multivariate model to quantify the FF in maternal plasma.

In this study, we aim to develop a gender-independent method, named Linkage Disequilibrium information-based cell-free Fetal DNA Fraction (LDFF), to accurately and robustly measure the FF in maternal plasma by only utilizing shallow-depth random sequencing (0.1x) of maternal plasma DNA. Compared with other existing methods, our LDFF method shows its distinct advantage in accurate determination of FF, even for low FF below 5%. Moreover, our results demonstrate that LDFF could be robustly applied to pregnancies with female fetus or pregnancies with complications. As this method is developed on the shallow-depth data, it could be readily integrated into the current NIPT practice to enhance the clinical performance.

## Results

### Positive correlation between regional LD-ratios and FFs

**Fig. 1:**
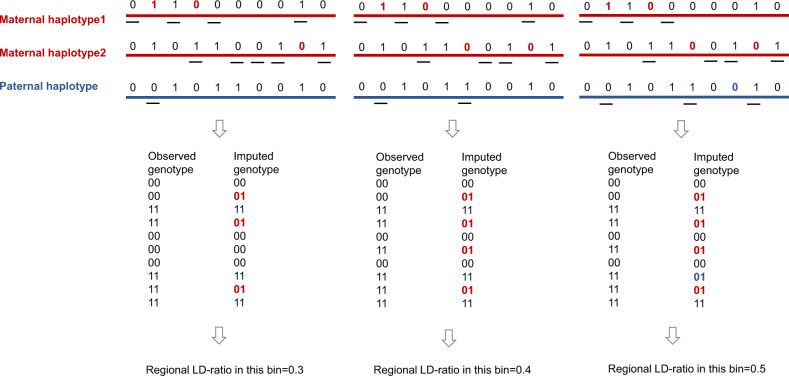
The schematic diagram of the LDFF method. Sequenced reads were aligned to the human reference genome(hg19). Regional LD-ratio was defined as the proportion of sites whose imputed genotypes were discordant with the corresponding observed genotypes in the specific regions of the autosomal chromosomes. Note: the short lines denote mapped reads. The observed genotypes were obtained by samtools mpileup. The imputed genotypes were detected by STITCH. 0 and 1 represent the reference base was concordant or discordant with the base of the read respectively.

**Fig. 2:**
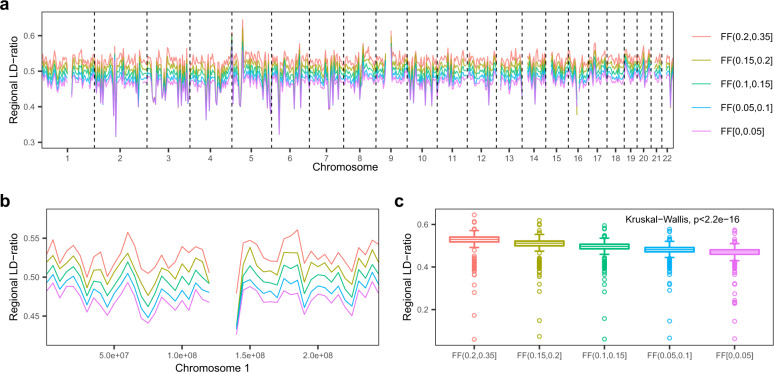
Regional LD-ratio profiles for the SNPs with minor allele frequency >0.2 in different FF groups. Training samples were partitioned into five groups according to chrY-based FF range. Average regional LD-ratios in each group on 22 autosomes (**a**) and chromosome 1 (**b**) were shown for each contiguous 5 Mb bins. **c** The differences of the regional LD-ratio between different FF groups. The FF values were estimated by chrY-based method. FF fetal fraction.

The detailed hypothesis and illustration are shown in principle of the LDFF in the method section and Fig. 1. To confirm this hypothesis, we grouped 10,000 male-bearing pregnancies in the training set into five groups by the FFs estimated by chromosome Y-based (chrY-based) method. From previous study^25^,we know that the fetal cfDNA is nonuniformly distributed across the whole genome, so we divided the genome into 5 Mb region, resulting in 567 regions across 22 autosomes (N-regions were excluded). The regional LD-ratio was calculated in each region for each sample. Averaging the regional LD-ratios of samples in each group per region, we observed that FFs were indeed linked to the average regional LD-ratios in each bin across 22 autosomes (Fig. 2a, b). We compared the distributions of the average regional LD-ratios of samples in each group across different regions by Kruskal–Wallis test and the results demonstrated values were significantly different among the five groups (*p*-value < 2.2e-16, Fig. 2c). Multiple comparison was further performed to determine difference of the regional LD-ratio levels between groups by Dunn’s test. Significant differences were observed between any two of the five groups (all the adjusted *p*-values < 3e-9, *p*-values were adjusted by the Benjamini–Hochberg method, Supplementary Table 1). We also analyzed the correlation between the FFs and regional LD-ratios in the training set. Taking the genome region chr1:1–5,000,000 for instance, the regional LD-ratios and the chrY-based FFs were significantly positively correlated (Pearson’s correlation coefficient, *R* = 0.174, *p*-value < 2.2e-16, Supplementary Fig. 1a). Significant positive correlations between the regional LD-ratios and chrY-based FFs were observed in 92.24% (523/567) of the tested genomic regions (adjusted *p*-value < 0.05, *p*-values were adjusted with the Benjamini–Hochberg method, Supplementary Fig. 1b, c). These results suggest that FFs are positively correlated with regional LD-ratios.

### LDFF model’s performance is improved by minor allele frequency filtering

Then, we constructed a multivariate linear regression model, named LDFF, to determine the relationship between the regional LD-ratios in each bin and the chrY-based FFs using the training data (Supplementary Fig. 2).

Due to the genetic difference, the genotypes of father and mother are more likely to be different at more common SNPs loci in the population. Previous studies^29^ found that variants with low minor allele frequency (MAF) are difficult to impute, as the imputation errors may increase with the decreased MAF. Thus, Pearson’s correlation coefficient (*R*) between the chrY-based FFs and predicted values and the mean absolute error in the training set without outliers were summarized using different MAF cutoff values (Supplementary Table 2). When the MAF cutoff was set to 0.2, the model (Supplementary Data 1) showed the optimal performance, with a correlation coefficient of 0.956 and the mean absolute error (MAE) of 0.01001. Therefore, 0.2 was finally chosen as the MAF filtering cutoff. Thus, SNPs loci with MAF larger than 0.2 were used in the calculation of regional LD-ratios, which included 2.88 million SNPs on the 19 autosomes in 1000 Genomes Project Phase 3 (1KGP3) East Asian population. The number of SNPs located in each 5M bin ranged from 35 to 211,187, with a median of 5534 (Supplementary Data 2).

### LDFF is accurate for predicting FF regardless of fetal gender and complications

**Fig. 3:**
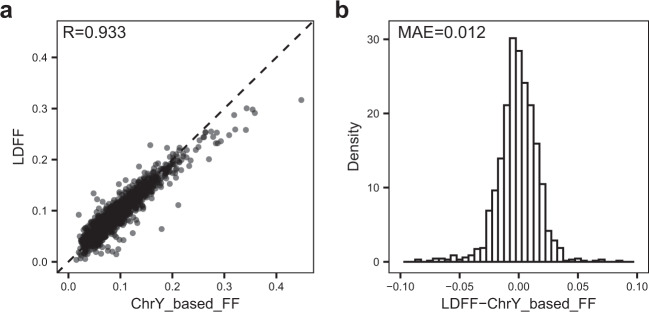
Performance of the LDFF in the male fetus testing set. **a** The FFs estimated by LDFF were highly positively correlated with the chrY-based FFs. **b** The normally distribution of errors centered around 0, showing the low mean absolute error (MAE) between FFs predicted by LDFF and FFs predicted by chrY-based method. MAE mean absolute error, FF fetal fraction, R Pearson’s correlation coefficient.

**Fig. 4:**
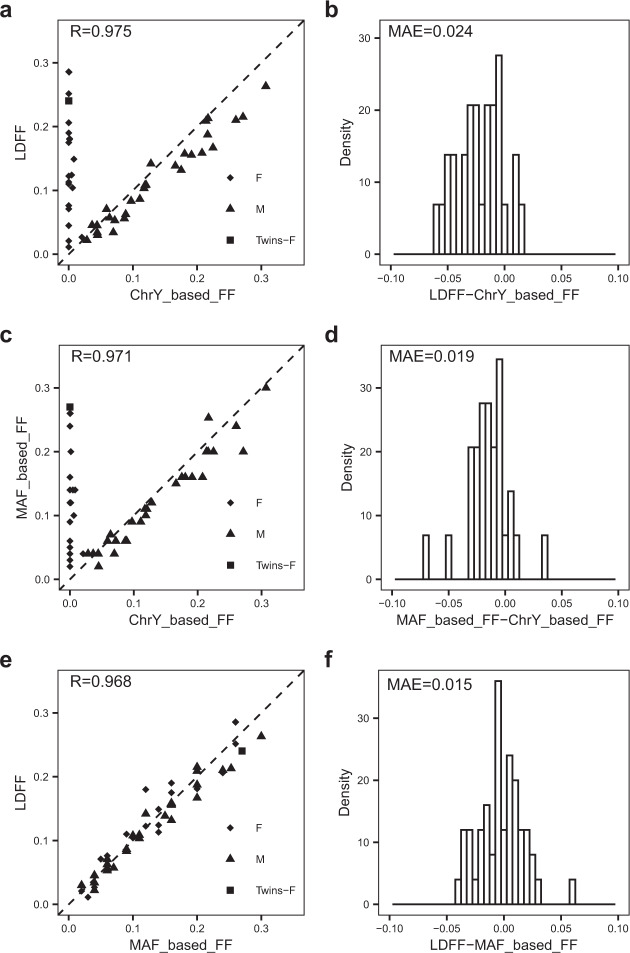
Performance of the LDFF in the external testing set. **a**, **b** The FFs estimated by LDFF were compared with the chrY-based FFs. **c**, **d** The FFs estimated by MAF-based method were compared with the chrY-based FFs. **e**, **f** The FFs estimated by LDFF were compared with the MAF-based FFs. The scatter plots reveal significantly positive correlation between two methods. The histograms are the distribution of differences between the FF values predicted by the two methods. For samples with female fetus, the minimum value of chrY-based FF was set to 0. F female, M male, Twins female-female identical twins, MAE mean absolute error, FF fetal fraction, R Pearson’s correlation coefficient.

To validate the performance, we applied this regression model to two datasets, including male fetus testing set and external test set. The FFs estimated by LDFF were then compared with expected FFs predicted by the chrY-based method or MAF-based method. FFs predicted by LDFF correlated strongly with the chrY-based FFs for the male fetus testing set (*R* = 0.933, *p*-value < 2.2e-16), and the corresponding MAE was 0.012 (Fig. 3). For male-bearing pregnancies in the external testing set, FFs from LDFF and chrY-based FFs were also highly correlated, with a Pearson’s correlation coefficient of 0.975 (*p*-value < 2.2e-16) and a MAE of 0.024 (Fig. 4a, b).

We further validated the accuracy of our method for pregnancies carrying either male or female fetus in external testing set. The high correlation (*R* = 0.971, *p*-value < 2.2e-16) and the low MAE (0.019) were observed between the chrY-based FFs and MAF-based FFs in pregnancies with male fetuses of the external testing set (Fig. 4c, d). Thus, we can conclude that MAF-based method has a high accuracy for FF estimation. Ultimately, we further validated the accuracy of our method for both male-bearing and female-bearing pregnancies in the external testing set, we found a significant correlation coefficient of 0.968 (*p*-value < 2.2e-16) and a MAE of 0.015 between LDFF and MAF-based method (Fig. 4e, f).

To test whether our method performs differently between pregnancies with female fetus and male fetus, we compared the MAF-based FFs and the FFs predicted by LDFF in 29 male-bearing pregnancies of the external testing set (Supplementary Fig. 3a, b, *R* = 0.981, MAE = 0.013) and 21 female-bearing pregnancies (Supplementary Fig. 3c, d, *R* = 0.961, MAE = 0.019), respectively. As expected, both groups showed a significant high correlation and a low MAE value, suggesting that LDFF is robust for predicting FF regardless of the fetal gender. To investigate whether the pregnancy complications alter the results, we calculated Pearson’s correlation coefficients and MAE values between the FF inferenced by LDFF and MAF-based FF in the samples with (Supplementary Fig. 3e, f, *R* = 0.959, MAE = 0.013) and without (Supplementary Fig. 3g, h, *R* = 0.969, MAE = 0.016) complications or β-thalassemia in the external testing set. The results demonstrated that the LDFF is able to give a reliable result for the samples with complications or β-thalassemia.

### LDFF outperforms existing methods in the estimation of FF

We further compared the performance of LDFF with other gender-independent FF estimation methods including SeqFF^25^ and PREFACE^30^ using the same testing sets (Table 1). These two methods were widely used for FF estimation by low-depth sequencing data and without the requirement of pre-knowledge of parental genotype information. Here, we used the pre-trained SeqFF model from Kim’s article^25^. The PREFACE model was trained with 3000 male-bearing pregnancies and 2000 female-bearing pregnancies in the first cohort. As compared with SeqFF and PREFACE, LDFF showed the highest correlations with chrY-based method or MAF-based method and the smallest MAE in all the test sets. We applied these methods to 185 samples with FFs <5% from the male fetus test set, our LDFF still had the highest Pearson’s correlation with chrY-based method and the smallest MAE (Supplementary Fig. 4). We also divided the samples in male fetus testing set into four groups according to different chrY-based FF and compared the performances of the three methods in each group. The correlation coefficients and the MAE values between chrY-based FF of these three methods demonstrated that in each FF interval, LDFF exhibited higher correlations and lower MAEs than other two methods (Supplementary Fig. 5). Therefore, our LDFF method exhibited a higher accuracy than other existing methods in the estimation of cell-free fetal DNA fraction.

**Table 1:** Performance of fetal fraction prediction by LDFF, SeqFF, and PREFACE methods.

		LDFF		SeqFF		PREFACE	
		Pearson’s correlation	MAE	Pearson’s correlation	MAE	Pearson’s correlation	MAE
chrY-based FF	Male fetus testing set (*n* = 1397)	0.933	0.012	0.910	0.016	0.922	0.015
	Pregnancies with FF <5% in male fetus testing set (*n* = 185)^a^	0.427	0.012	0.300	0.015	0.386	0.013
	Pregnancies with male fetus in external testing set (*n* = 29)	0.975	0.024	0.948	0.024	0.920	0.026
MAF-based FF	External testing set (*n* = 50)	0.968	0.015	0.895	0.028	0.915	0.031

*MAE* mean absolute error.

^a^Among male fetus testing set, 185 samples with fetal fraction <5% were selected.

## Discussion

The methodology described in this study for the detection of fetal fraction relies on a simple and fundamental assumption that the fetal cfDNA has different alleles from the mother’s and this allele information is mixed in maternal plasma. Moreover, this difference can be quantified from the level of discrepancy between observed genotypes and imputed genotypes based on the known haplotypes in a population, which is proportional to the fetal fraction. We have tested this hypothesis over a set of 10,000 pregnancies bearing a male fetus and have demonstrated it to be consistent with these assumptions. The regional LD-ratios were found to be positively correlated with the FFs in most of genomic regions, although the correlation coefficients were relatively low, with a median of 0.106 (interquartile range, 0.089–0.120, Supplementary Fig. 1b). However, high dimensional machine learning approach can uncover the relationships in a large cohort of samples. A weighted multivariate model was applied to determine the relationship between FFs and regional LD-ratios. Furthermore, the accuracy of the LDFF method was verified in two cohorts. The FFs estimated by the LDFF method were significantly correlated with those predicted by the chrY-based method in two male-bearing pregnancies data sets and those calculated by the MAF-based method in pregnancies either with male or female fetus set. By comparing the performance of LDFF with other FF estimation methods, we showed that LDFF has higher accuracy than the other methods.

The core theory of our innovation is that existing fetal alleles are different from the maternal alleles in the low coverage sequencing data of maternal plasma. Thus, parts of these loci might be rectified by imputation process and this information could be used for FF estimation. Several methods have been previously established to estimate FF values using the fetal-specific alleles. However, the limitation for these approaches is maternal genotype^22,31^ or even parental genotype^5,21^ information needs to be identified by microarray-based genotyping technologies. Therefore, these methods require extra laboratory tests, causing increased cost to patients. It is necessary to determine the FF directly from the same next generation sequencing (NGS) data that used for NIPT. Our algorithm was developed on the shallow-depth data without requiring the prior knowledge of fetal gender and parental genotype information, thus expensive paired-end sequencing or additional laboratory assay is not required. Therefore, this method can be easily integrated into the current NIPT protocol in a cost-effective manner. Furthermore, we demonstrated that LDFF was capable of accurately estimating FF independently of fetal gender.

Accurate measurement of fetal fraction in maternal plasma is essential to the NIPT practice, bringing several benefits. Firstly, if the FF is below the limit of detection, a “no call” report is an effective way to reduce false negatives in fetal trisomy screening. Secondly, studies suggest pregnancies with small FFs may increase the risk of fetal aneuploidy^10,32^. Therefore, samples with low FF can be re-sequenced at an increased sequencing depth to achieve sufficient power for the fetal aneuploidy determination. Thirdly, in the prediction of monogenic disease, the FF is also a key parameter^5,6^ to determine the statistical thresholds to ensure the statistical significance. Lastly, when FF is accurately known, a more powerful test to screen for fetal trisomy can be developed^28,33^ and the fetal sex determination in twin pregnancies^34,35^ also becomes a much simpler problem. The ability of measuring FF with high accuracy would make LDFF serve as a valuable tool to improve NIPT performance.

However, two kinds of maternal characteristics might affect the fetal fraction estimation of LDFF. First, when there are more than three haplotypes exist in maternal plasma per genomic region, including maternal chromosomal mosaicism, maternal chromosomal aneuploidy and transplants of donor tissues, the regional LD-ratio may be skewed. And also, when a pregnant woman harbors a malignant tumor, the apoptotic cell-free tumor DNA can shed into the circulation, then an increased tendency of genomic abnormalities including long-term copy-number variation (CNV) and mutations may be detected by whole-genome sequencing and skew the regional LD-ratio. All these maternal incidental biological causes may result in an altered fetal fraction when using LDFF for FF estimation, and false results may also occur in NIPT. Second, the benign long-term copy-number variation (>5 M) in maternal genome is an extra factor that may affect the fetal fraction estimation. However, as the number of regions (521) and the number of SNP loci (~2 million) used for estimating FF are large enough, the frequency of CNV in the human genome is relatively low^36^, especially for the large CNVs, the specific bias caused by the certain CNV would contribute little to the multiple linear regression model.

As the participate in this study all come from Chinese population, thus haplotype and allele frequency information from the Chinese population in the 1000 Genomes Project Phase 3(1KGP3) were taken as the reference panel. When the imputation is performed with the reference panel including the same population or closely related populations, the accuracy of the SNP imputation can be high^37^. So, for other populations, the imputation reference panel should be changed accordingly to ensure high imputation accuracy. In the meantime, the SNPs with MAF >0.2 were used in the regional LD-ratio calculation in this study. However, the common SNPs might not be consistent in different popualations^38^, the training process would need to be repeated to obtain a new set of parameters for the specific population. Once the model is well-trained on that population, it could be readily applied to any test data, as long as they are generated from the same population.

The time consumption of LDFF and other two commonly used methods are also shown in Supplementary Table 3. Comparing with SeqFF and PREFACE, our method does require more running time due to the time-consuming step of SNP imputation. Large scale of training samples increases the model accuracy, meanwhile, the training time is increased. Moreover, we calculated the regional LD-ratios for the whole genome. Advanced machine learning model could be used to select sub-domains of the genome, which could improve the accuracy and reduce the computational time. It is necessary to optimize the imputation process to reduce the time required. Furthermore, widely used hardware such as GPU and large-scale computing resources can be used to further reduce the running time.

In summary, the LDFF demonstrated in this study is highly accurate and gender-independent for the prediction of FF, showing its utility in noninvasive prenatal testing. Furthermore, as this method is developed on shallow-depth sequencing data, it can be easily incorporated into the clinical protocols currently used by laboratories offering sequencing-based NIPT service.

## Methods

### Sample collection and ethical statement

Two independent cohorts of samples were collected. The first cohort with singleton pregnancies was randomly selected from a previous study^39^, in which pregnant women undergoing NIPTs at BGI Clinical Laboratories were recruited. The second cohort from another study comprised 50 samples including 29 pregnancy samples with male fetuses, 20 samples with female fetuses, and 1 sample with female–female monozygotic twins. All participants provided written informed consent and granted permission to anonymously use NIPT sequencing data for research purpose. This retrospective study was in strict compliance with regulations regarding ethical considerations and personal data protection, and it was approved by the Institutional Review Board of BGI.

### Sequencing

For each participant in the first cohort, 5 ml of peripheral blood was collected for the NIPT test. Briefly, plasma was extracted from whole blood within 8 h of blood collection. After library construction and sample quality control, sequencing was conducted on the Illumina Hiseq 2000 platform to produce 3.72–13.34 million 49 bp single-end reads^9^. Each plasma sample from the second cohort was sequenced on the DNBSEQ platform using the 100 bp paired-end mode to produce ~3900 million reads. Then we down-sampled the sequencing data to 7 million 49 bp single-end reads from the second cohort using the mate-pair 1 reads of each sample.

### Bioinformatics analyses

Low quality reads with >30% low quality bases (*Q* < 20) or N bases were removed by SOAPnuke. After data filtering, the cleaned reads were aligned to the human reference genome(hg19) by Burrows-Wheeler Aligner(bwa)^40^. The original (observed) genotypes were generated by samtools^41^ mpileup and stored in VCF format. The imputed genotypes were detected by STITCH (version v1.5.3.0008)^42^ in a 5 Mb window with 250 kb buffer. And this method was confirmed to have no batch effect. We took genetic information from the Chinese population (CHB + CHS + CDX, *n* = 301) in the 1KGP3 as reference panel. The imputed loci were composed of 7.89 million known polymorphic sites in 22 autosomes, with allele frequency ≥0.01 in 1KGP3 East Asian.

### Training data and Testing data

Two independent cohorts of samples were used in this study. The first cohort with singleton pregnancies was selected from a previous publication^39^, in which all participants undergoing NIPT were recruited. The training set is defined as the samples used to train the statistical model. Ten thousand pregnant samples with male fetuses in the first cohort were randomly selected and assigned to the training set, including 20 fetuses with confirmed trisomy for chromosome 13, 18, or 21 by chorionic villus sampling or amniocentesis.

To evaluate the performance of our FF estimation method, we designed two testing sets containing one set of pregnancies carrying male fetus from the first cohort and the external testing set from the second cohort. The male fetus testing set contains additional 1397 male-bearing pregnancies randomly selected from the first cohort which are different from the samples in the training set. Among these samples, three were from pregnancies with trisomic fetuses of chromosome 21(Supplementary Table 4). There is no significant difference between the FF distribution in the male fetus testing set and training set (*p*-value = 0.2897, Wilcoxon rank-sum test).

The external testing set comprised 50 additional pregnancies including 29 samples with male fetuses, 20 samples with female fetuses, and 1 sample with female-female monozygotic twins. Among these samples, seven pregnancies had pregnancy complications (gestational diabetes mellitus, premature delivery, preeclampsia, or intrahepatic cholestasis of pregnancy), two pregnancies had β-thalassemia. The samples in this cohort were previously collected for monogenic disease research with the raw sequencing depth of nearly 130x. The high depth sequencing reads were down-sampled to similar sequencing depth with NIPT (7 million reads) for all samples, which were used as the external testing set to independently verify the model’s accuracy and robustness in the inference of FF.

Clinical characteristics of participants of different datasets were described in Supplementary Table 4, including gestational week, maternal age and body mass index (BMI), karyotype, and complications. The total read number and the mapped coverage for each group were also shown in the Supplementary Table 4.

### LDFF method

Because of the shallow sequencing depth, most of the SNP loci were covered by only one read. Therefore, the genotypes in certain SNP sites might be wrongly exhibited as homozygous by samtools^41^ mpileup procedure due to the limited number of reads covering these SNP loci. Genotype imputation is a commonly used statistical technique to infer the missing data or correct low probability genotype. This technique strongly relies on linkage disequilibrium (LD) or allelic association through comparison with known haplotypes in a population, for instance from the 1KGP3 or the HapMap. Genotype imputation algorithms assume the analyzed samples are diploid. Therefore, when there are genotypes from more than two haplotypes that contradict with this hypothesis, these loci are regarded as the wrong locus. In fact, pregnant women’s plasma contains three haplotypes per genomic region including two haplotypes from the mother and one fetal haplotype inherited from the father. Therefore, in the process of imputation, there was a certain probability that the site on one of three haplotypes was considered as the wrong locus, which was correlated with the fetal cfDNA concentration in maternal plasma. When genotype imputation was performed, part of the un-sequenced maternal alleles (red sites) and the fetal alleles inherited from father (blue sites) could be inferred (Fig. 1). The ratio of sites whose imputed genotypes were discordant with the observed genotypes across the specific regions can be calculated, namely regional LD-ratio. However, a small proportion of the inconsistent genotypes could be caused by the sequencing errors in maternal plasma and/or the wrong imputation. Assuming these kinds of inconsistences were relatively constant across different samples, we hypothesized that the FF would be proportional to regional LD-ratio.

A multivariate linear regression model was employed to predict FF. The response variable was chrY-based FF, which can be directly calculated by previously described method^14^. We then divided the hg19 autosomes into contiguous 5 Mb regions. The regions located on chromosome 13, 18, 21, X, and Y were excluded from our model to avoid over-fitting due to the fetal aneuploidy or fetal gender. Hence, the final region included 521 adjacent, non-overlapping 5 Mb bins on 19 autosomes (N-regions were excluded). The start and end positions of each bin were shown in Supplementary Data 1. We defined the regional LD-ratio as the ratio of candidate sites changed by genotype imputation from the observed genotype generated by samtools in each bin. To take into account bias in sequencing characteristics of the genome, we added total genome coverage, coverage of the reads with a mapping quality (MQ) score >0 and polymerase chain reaction (PCR) duplication rate as the confounders to the model. The coefficients of the linear regression model can be determined by the chrY-based FFs and the predictor variables, including 521 regional LD-ratio values and all the confounders, using the training data.

To achieve the best FF inference performance, we performed MAF filtering and removed all the SNPs with MAF values less than the cutoff to calculate regional LD-ratios. To determine which MAF cutoff should be used in MAF filtering, different MAF filtering values (0.15, 0.2, 0.25, 0.3) in 1KGP3 East Asian (CHB + CHS + CDX + JPT + KHV, *n* = 504) were analyzed. We built several models and compared the Pearson’s correlation and MAE between the chrY-based FFs and predicted FFs using different MAF filtering cutoffs. Finally, MAF values were set as the cutoff values according to the following criterion. The MAF value showed the highest Pearson’s correlation between the chrY-based FFs and the predicted values in the training set without outliers.

To avoid over-fitting in the training set, a custom R script was used to detect outliers in the training set. The samples were considered as outliers if it met any of the following criteria: (1) samples show maximum absolute residual values; (2) samples have maximum absolute studentized residual values (3) samples show maximum absolute standardized residual values; (4) samples have the diagonal elements of the hat matrix $> 2\frac{{p + 1}}{n}$, *p* is the number of coefficients in the regression model; *n*, the number of samples; (5) DFFITS^43^ $> 2\sqrt {\frac{{p + 1}}{n}}$, *p* is the number of coefficients in the regression model; *n*, the number of samples; (6) samples with the maximum Cook’s distances;^44^ and (7) samples with the maximum COVRATIO distance to 1.

Once the model parameters were determined, the fetal fraction for other data sets could be estimated by adding the regional LD-ratio in each bin and all the confounders after weighing them by their respective coefficient. The workflow of LDFF consisted of four steps (Supplementary Fig. 2). First, the regional LD-ratios, the genome coverage, coverage of the reads with a MQ score >0 and PCR duplication rate were calculated for the 521 genomic regions. Several multivariate regression models were generated with different MAF filtering cutoffs using all training samples. Then, the outliers in the training samples in different MAF filtering models were identified and removed from the corresponding model to avoid over-fitting. Finally, the model which had the best accuracy was selected as the final model, the MAF filtering cutoff was selected accordingly.

### Chromosome Y-based method

The referenced FF in male fetuses can be calculated by the method described in Hudecova et al.^14^. In brief, the reads mapped to chromosome Y(chrY) in maternal plasma consisted of the amount of chromosome Y sequences contributed by the male fetus and sequences originated from the maternal background DNA that were incorrectly mapped to chromosome Y. Thus, the FF(*F*) was estimated using the following formula:

1 $$\% {{chrY}} = \left( {{{male}}\% {{chr Y}} \times {F}} \right) + {{female}}\% {{chrY}} \times \left( {1 - F} \right),$$

2 $${\mathrm{where}}\,F = \frac{{{\%}{{chrY}} - {{female}}\% {{chrY}}}}{{{{male}}\% {{chrY}} - {{female}}\% {{chrY}}}}$$

*Male %chrY, female %chrY* represented the mean fraction of chrY reads of plasma samples obtained from adult male individuals and from pregnancies bearing euploid female fetuses respectively.

### MAF-based method

After alignment, high coverage BAM files from the second cohort were piled up by samtools mpileup and minor allele frequency (MAF) was calculated for each locus. The MAF distribution is known to consist of reads from pregnant women and fetuses. Assume that FF is *F*, so the peak of the distribution corresponds to the MAF of *F*/2 for loci that are homozygous for maternal genotype and heterozygous for fetal genotype. Therefore, for MAF ∈ [0, 0.25], its distribution was regarded as a mixed distribution of several normal distributions, and the peak of the MAF distribution was *F*/2. Finally, the FF of the plasma cfDNA was obtained.

### Reporting summary

Further information on research design is available in the Nature Research Reporting Summary linked to this article.

## Supplementary information

Supplementary information

Supplementary Data1

Supplementary Data 2

Reporting Summary

## Data Availability

The cfDNA sequencing data of fifty pregnancies in the second cohort have been deposited in the Sequence Read Archive (SRA) with the accession number PRJNA756388. The release of the data was approved by the Ministry of Science and Technology (MOST) of China (Project ID:2021BAT2647).

## Code availability

LDFF was implemented using Perl (https://www.perl.org/) and R (https://www.r-project.org/) languages. Perl script was used to calculate the regional LD-ratio and R language was used to construct the linear regression model. The source codes were available at https://github.com/jujia-BGI/LDFF_V1.
