# Supplementary material for: Estimation of cell-free fetal DNA fraction from maternal plasma based on linkage disequilibrium information
Source: NPJ Genom Med. 2021 Oct 12;6:85. doi: 10.1038/s41525-021-00247-z (PMC8511193; doi:10.1038/s41525-021-00247-z)
Supplement: Supplementary file 1 — Supplementary information [file 41525_2021_247_MOESM1_ESM.pdf]

Supplementary information:

Estimation of cell-free fetal DNA fraction from maternal plasma based on linkage disequilibrium information

In this PDF the Supplementary Figures (1-5) and Supplementary Tables (1-4) are presented. The Supplementary Data (1,2) are presented in the Excel files. The titles are shown as follows.

Supplementary Data 1. The coefficients of the multivariate linear regression model

Supplementary Data 2. Number of SNPs in each region (The SNPs MAF information is from 1000 GP East Asian)

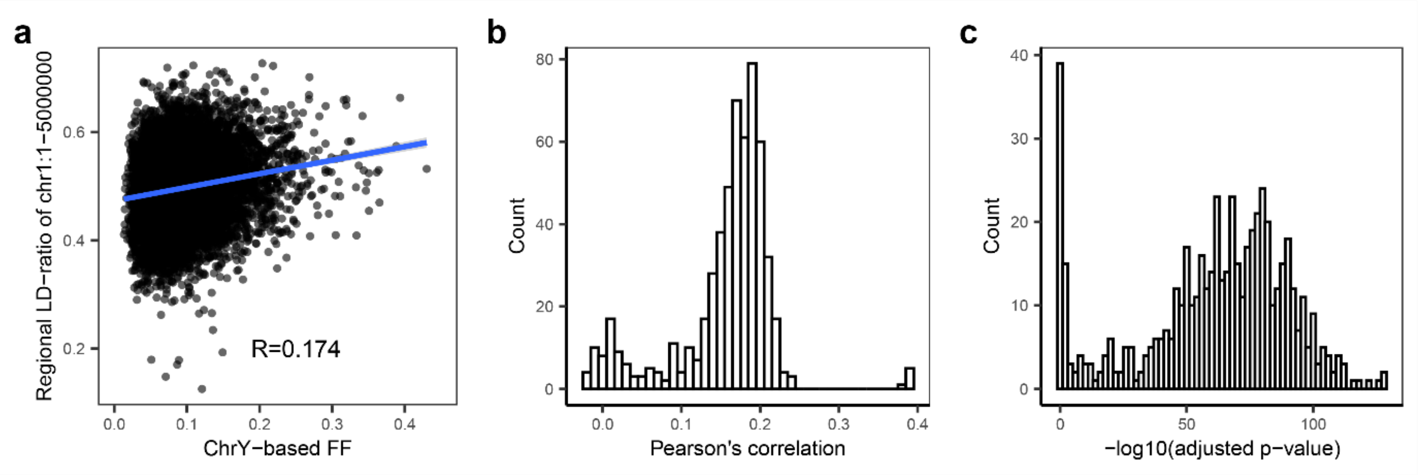

Supplementary Figure 1. Pearson's correlation between regional LD-ratios and the expected FFs inferred from chrY-based method. **a** An example scatter plot indicates regional LD-ratios positively (Pearson's correlation coefficient, p-value=6.49E-69) correlated with chrY-based FFs in the region of chr1:1-5000000. The distribution of Pearson's correlations between regional LD-ratios and chrY-based FFs for each bin (**b**) and the responding adjusted p-values (**c**).

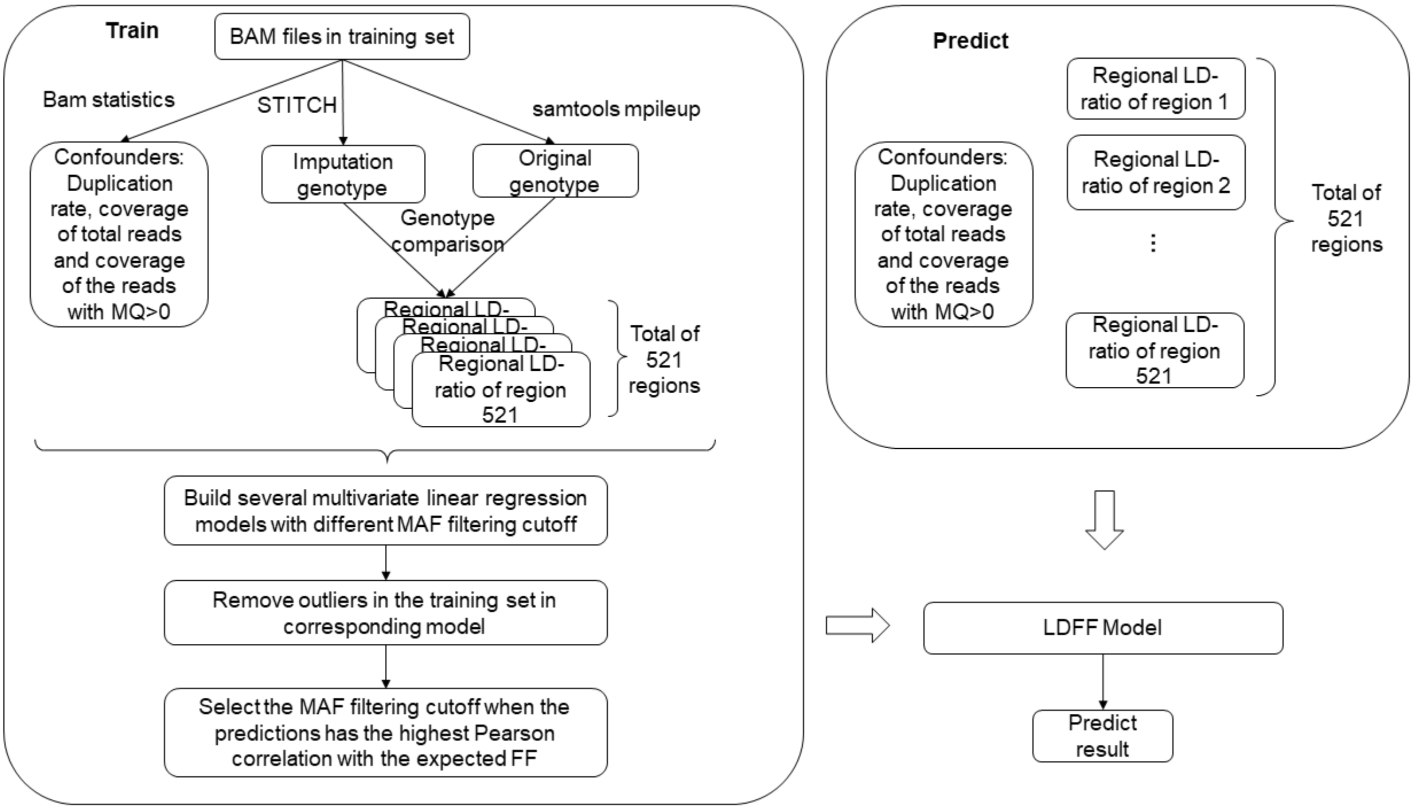

Supplementary Figure 2. Schematic representation of the LDFF method. The workflow consists of four processes. First, the regional LD-ratios, the genome coverage, coverage of the reads with a MQ score>0 and PCR duplication rate were calculated for the 521 genomic regions. Second, several multivariate regression models were generated with different MAF filtering cutoffs using all training samples. Then, the outliers in different MAF filtering models were identified and removed from the corresponding model to avoid over-fitting. Multivariate regression models were rebuilt using the remaining samples. Finally, the model which had the best accuracy was selected as the final model, the MAF filtering cutoff was selected accordingly.

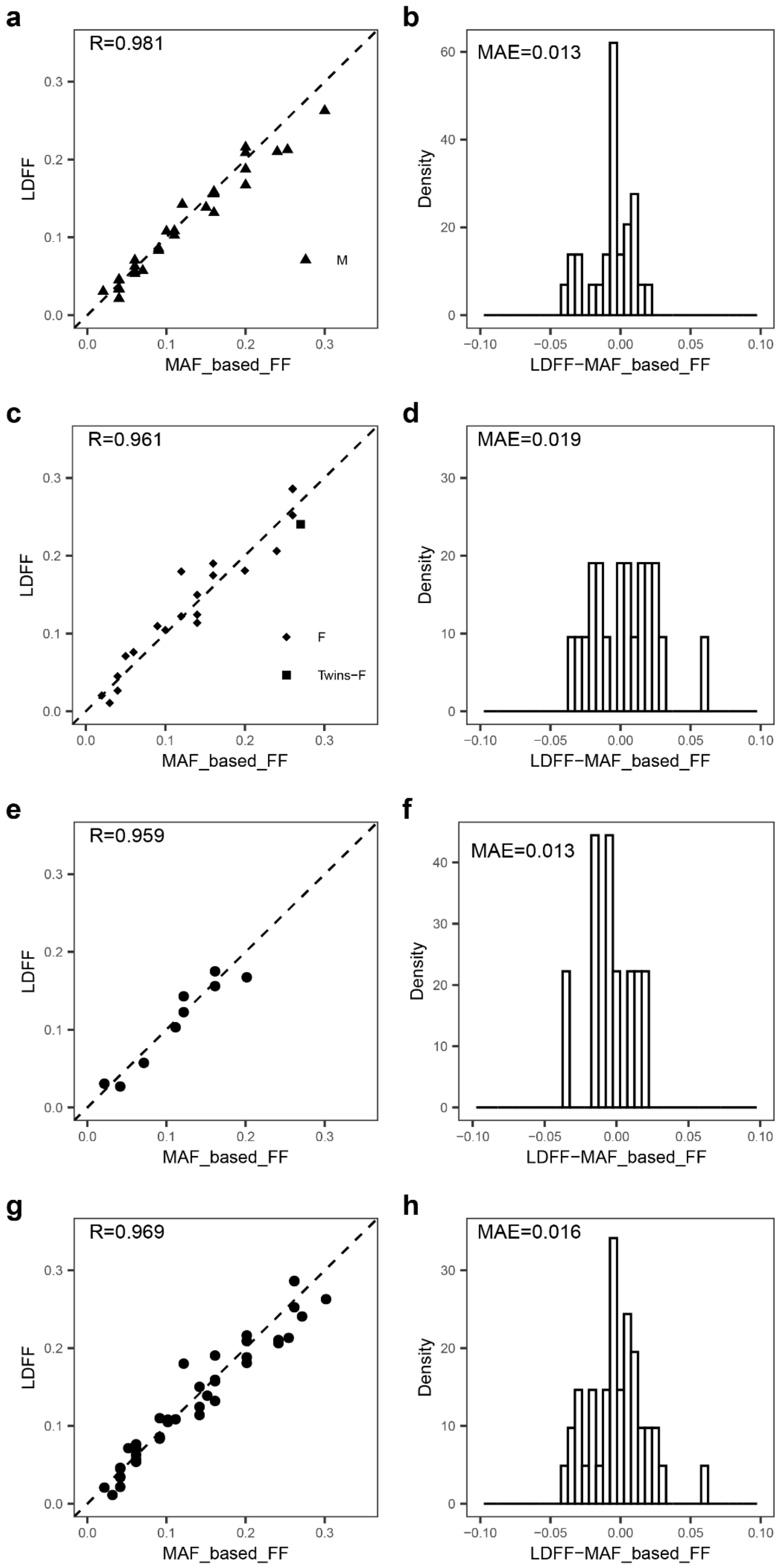

a
R=0.981
M
LDFF
MAF_based_FF
b
MAE=0.013
Density
LDFF−MAF_based_FF
c
R=0.961
F
Twins−F
LDFF
MAF_based_FF
d
MAE=0.019
Density
LDFF−MAF_based_FF
e
R=0.959
LDFF
MAF_based_FF
f
MAE=0.013
Density
LDFF−MAF_based_FF
g
R=0.969
LDFF
MAF_based_FF
h
MAE=0.016
Density
LDFF−MAF_based_FF

Supplementary Figure 3. Performance of the LDFF in the external testing set. (**a, b**) The FFs estimated by LDFF were compared with the MAF-based FFs in the pregnancies with male fetus of the external testing set. (**c, d**) The FFs estimated by LDFF were compared with the MAF-based FFs in the pregnancies with female fetus of the external testing set. (**e, f**) The FFs estimated by LDFF were compared with the MAF-based FFs in the pregnancies with complications (such as gestational diabetes mellitus, intrahepatic cholestasis of pregnancy, preeclampsia, et al) or β-thalassemia of the external testing set. (**g, h**) The FFs estimated by LDFF were compared with the MAF-based FFs in the healthy pregnant women without complications. Abbreviations: F, female; M, male; Twins, female-female identical twins; MAE, mean absolute error; FF, fetal fraction; R, Pearson's correlation coefficient

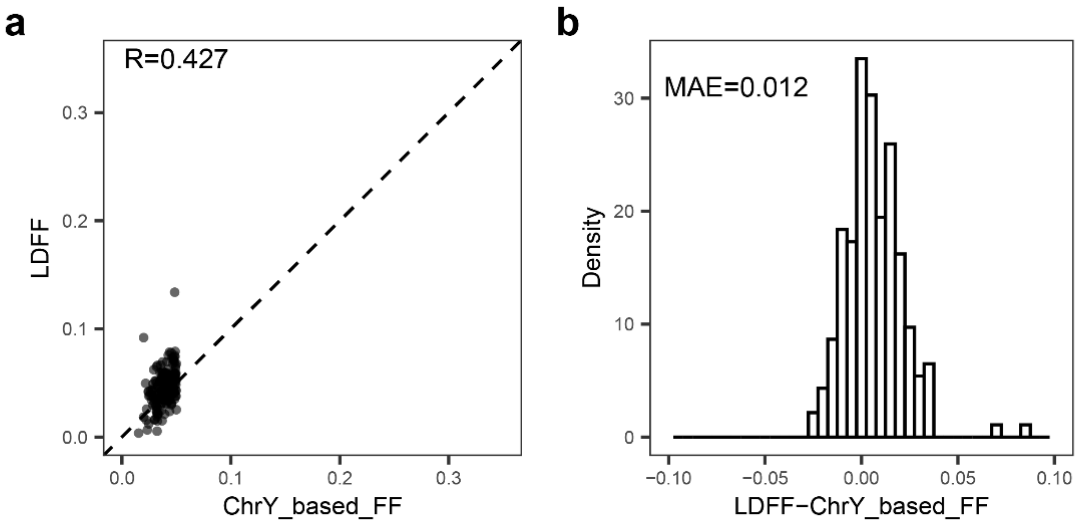

Supplementary Figure 4. Performance of the LDFF in the male fetus testing set with FF less than 5%. **a** FFs estimated by LDFF were compared with the chrY-based FFs. **b** The distribution of the differences between FFs inferred by the LDFF and FFs inferred by the chrY-based method. Abbreviations: MAE, mean absolute error; FF, fetal fraction; R, Pearson's correlation coefficient

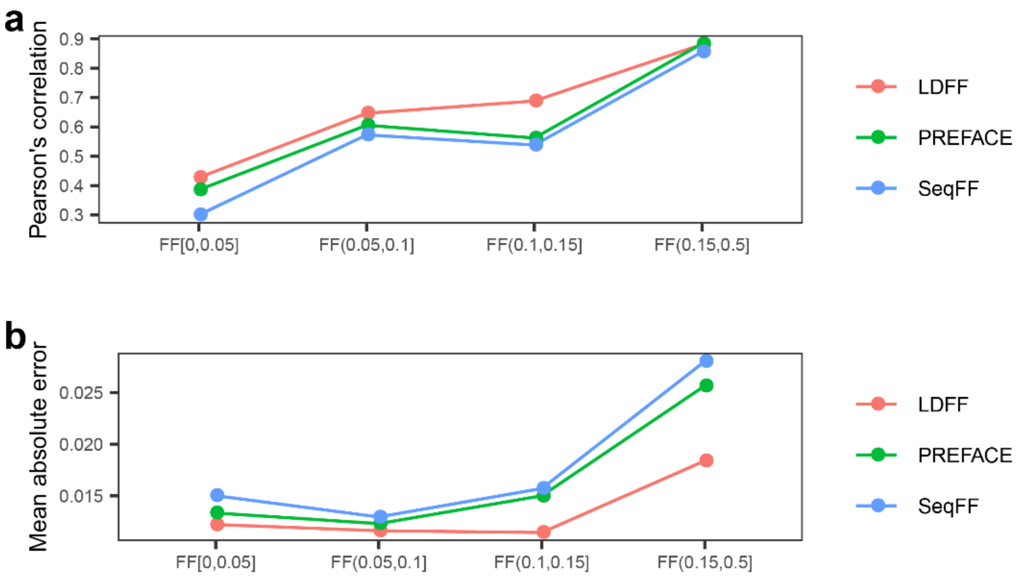

Supplementary Figure 5. Comparison of three FF estimation methods in subgroups of the male testing set stratified by different FF intervals in male testing set. **a** The differences in Pearson's correlation coefficients among the three methods in subgroups of the male testing set stratified by different FF intervals. **b** The differences in mean absolute errors among the three methods in subgroups of the male testing set stratified by different FF intervals. The number of samples are 185, 685, 366, 161 for the subgroups of FF[0,0.05], FF[0.05,0.1], FF[0.1,0.15]), FF[0.15,0.5], respectively.

Supplementary Table 1. Dunn test for Kruskal-Wallis multiple comparison of the average regional LD-ratios of samples in each group across different regions

Comparison	Z	P.unadjust	P.adjust[1]
FF[2](0.05,0.1] - FF(0.1,0.15]	-8.719	2.80E-18	3.50E-18
FF(0.05,0.1] - FF(0.15,0.2]	-17.448	3.57E-68	8.93E-68
FF(0.1,0.15] - FF(0.15,0.2]	-8.729	2.58E-18	3.68E-18
FF(0.05,0.1] - FF(0.2,0.35]	-25.758	2.62E-146	1.31E-145
FF(0.1,0.15] - FF(0.2,0.35]	-17.039	4.23E-65	8.47E-65
FF(0.15,0.2] - FF(0.2,0.35]	-8.310	9.56E-17	1.06E-16
FF(0.05,0.1] - FF[0,0.05]	5.942	2.82E-09	2.82E-09
FF(0.1,0.15] - FF[0,0.05]	14.661	1.15E-48	1.91E-48
FF(0.15,0.2] - FF[0,0.05]	23.390	5.44E-121	1.81E-120
FF(0.2,0.35] - FF[0,0.05]	31.700	1.57E-220	1.57E-219

[1]p-values were adjusted with the Benjamini-Hochberg method.

[2]The FF values were calculated by chrY-based method.

Supplementary Table 2. The correlation between the chrY-based FF and FF values predict by LDFF with different minor allele frequency (MAF) filtering cutoff in training set

	**Pearson's correlation**	**MAE**
MAF[1]> 0.15	0.954	0.01015
**MAF > 0.2**	**0.956**	**0.01001**
MAF > 0.25	0.955	0.01002
MAF >0.3	0.953	0.01025

[1]The MAF information is from 1000 GP East Asian

Supplementary Table 3. Time consumption

	No. of Sample	LDFF	SeqFF	PREFACE
Time(min)	10	420	32	15
	100	1370	320	150
	1000	15720	3200	1500

Supplementary Table 4. Basic characteristics of different pregnant women dataset[1]

	**Training set (n=10000)**	**Male fetus testing set (n=1397)**	**External testing set (n=50)**
Fetal fraction[2]	8.5% (1.3%-48%)	8.6% (1.5%-44.8%)	12% (2%-30%)
Gestational week at blood sampling	18(10-40,3 unknown)	18(10-33,1 unknown)	25(12-37, 1 unknown)
Maternal age (year)	30(15-49,64unknown)	31(17-45,10 unknown)	30(22-34)
BMI[3]	22.30(15.60-40，7242unknown)	22.1(15.6-39.50, 1005unknown)	19.70(9.89-26.08, 1 unknown)
Karyotype			
Trisomy 21	16	3	0
Trisomy 18	3	0	0
Trisomy 13	1	0	0
Complications or disease	unknown	unknown	9
Gestational diabetes mellitus	/	/	4
Premature delivery	/	/	1
Intrahepatic cholestasis of pregnancy	/	/	1
Preeclampsia	/	/	1
β-thalassemia	/	/	2
Total reads (Million)	7.16(3.72-13.17)	7.22(4.52-13.34)	7(7-7)
Mapped coverage	0.111(0.057-0.204)	0.112(0.087-0.206)	0.108(0.096-0.109)

[1] Values are median(range)

[2] FFs of Training set and male fetus testing set are based on chromosome-Y method; FFs of the external testing set are based on MAF method

[3] BMI, body mass index
