# Supplementary material for: Estimation of cell-free fetal DNA fraction from maternal plasma based on linkage disequilibrium information
Source: NPJ Genom Med. 2021 Oct 12;6:85. doi: 10.1038/s41525-021-00247-z (PMC8511193; doi:10.1038/s41525-021-00247-z)
Supplement: Supplementary file 4 — Reporting Summary [file 41525_2021_247_MOESM4_ESM.pdf]

nature research

Corresponding author(s): Xin Jin
NPJGENMED-00733R1

Last updated by author(s): Sep 1, 2021

Reporting Summary

Nature Research wishes to improve the reproducibility of the work that we publish. This form provides structure for consistency and transparency in reporting. For further information on Nature Research policies, see our Editorial Policies and the Editorial Policy Checklist.

Statistics

For all statistical analyses, confirm that the following items are present in the figure legend, table legend, main text, or Methods section.

n/a	Confirmed	
☐	☒	The exact sample size (*n*) for each experimental group/condition, given as a discrete number and unit of measurement
☐	☒	A statement on whether measurements were taken from distinct samples or whether the same sample was measured repeatedly
☐	☒	The statistical test(s) used AND whether they are one- or two-sided<br>*Only common tests should be described solely by name; describe more complex techniques in the Methods section.*
☐	☒	A description of all covariates tested
☐	☒	A description of any assumptions or corrections, such as tests of normality and adjustment for multiple comparisons
☐	☒	A full description of the statistical parameters including central tendency (e.g. means) or other basic estimates (e.g. regression coefficient) AND variation (e.g. standard deviation) or associated estimates of uncertainty (e.g. confidence intervals)
☐	☒	For null hypothesis testing, the test statistic (e.g. *F*, *t*, *r*) with confidence intervals, effect sizes, degrees of freedom and *P* value noted<br>*Give P values as exact values whenever suitable.*
☒	☐	For Bayesian analysis, information on the choice of priors and Markov chain Monte Carlo settings
☒	☐	For hierarchical and complex designs, identification of the appropriate level for tests and full reporting of outcomes
☐	☒	Estimates of effect sizes (e.g. Cohen's *d*, Pearson's *r*), indicating how they were calculated

Our web collection on statistics for biologists contains articles on many of the points above.

Software and code

Policy information about availability of computer code

Data collection	no software was used
Data analysis	Burrows-Wheeler Aligner(Version: 0.7.15-r1140)<br>samtools(Version: 1.9)<br>STITCH (Version v1.5.3.0008)<br>LDFF_V1(https://github.com/jujia-BGI/LDFF_V1)<br>SeqFF<br>PREFACE

For manuscripts utilizing custom algorithms or software that are central to the research but not yet described in published literature, software must be made available to editors and reviewers. We strongly encourage code deposition in a community repository (e.g. GitHub). See the Nature Research guidelines for submitting code & software for further information.

Data

Policy information about availability of data

All manuscripts must include a data availability statement. This statement should provide the following information, where applicable:

- Accession codes, unique identifiers, or web links for publicly available datasets
- A list of figures that have associated raw data
- A description of any restrictions on data availability

The cfDNA sequencing data of fifty pregnancies in the second cohort have been deposited in the Sequence Read Archive (SRA) with the accession number PRJNA756388. The release of the data was approved by the Ministry of Science and Technology (MOST) of China (Project ID:2021BAT2647).

Field-specific reporting

Please select the one below that is the best fit for your research. If you are not sure, read the appropriate sections before making your selection.

☒ Life sciences ☐ Behavioural & social sciences ☐ Ecological, evolutionary & environmental sciences

For a reference copy of the document with all sections, see nature.com/documents/nr-reporting-summary-flat.pdf

Life sciences study design

All studies must disclose on these points even when the disclosure is negative.

Sample size	In total 1447 samples from two data sets were used as the test data, including male-bearing pregnancies, female-bearing pregnancies, pregnancies with low fetal fractions(less than 0.05) and fetuses with confirmed trisomy. The test set include most of clinical scenario of NIPT application.
Data exclusions	No data were excluded.
Replication	In this study our LDFF model has been verified in different data sets and the performance is proved to be robust.
Randomization	YES, the samples are all randomly allocated into groups.
Blinding	YES

Reporting for specific materials, systems and methods

We require information from authors about some types of materials, experimental systems and methods used in many studies. Here, indicate whether each material, system or method listed is relevant to your study. If you are not sure if a list item applies to your research, read the appropriate section before selecting a response.

Materials & experimental systems

n/a	Involved in the study	
☒	☐	Antibodies
☒	☐	Eukaryotic cell lines
☒	☐	Palaeontology and archaeology
☒	☐	Animals and other organisms
☐	☒	Human research participants
☒	☐	Clinical data
☒	☐	Dual use research of concern

Methods

n/a	Involved in the study	
☒	☐	ChIP-seq
☒	☐	Flow cytometry
☒	☐	MRI-based neuroimaging

Human research participants

Policy information about studies involving human research participants

Population characteristics	All participants are pregnancies, including normal, bearing fetuses with trisomy, pregnancy complications.
Recruitment	Two independent cohorts of samples were collected. The first cohort with singleton pregnancies were randomly selected from a previous study(Liu et al., 2018, Cell 175, 347–359) ,in which pregnant women undergoing NIPTs at BGI Clinical Laboratories were recruited. The second cohort were recruited by Department of Fetal Medicine and Prenatal Diagnosis, the Guangdong Women and Children's Hospital and Health Institute,China.
Ethics oversight	This study was approved by the Institutional Review Board of BGI.

Note that full information on the approval of the study protocol must also be provided in the manuscript.
